# Sex difference in the associations among liver function parameters with incident diabetes mellitus in a large Taiwanese population follow-up study

**DOI:** 10.3389/fpubh.2022.1081374

**Published:** 2023-01-04

**Authors:** Yi-Kong Chen, Pei-Yu Wu, Jiun-Chi Huang, Szu-Chia Chen, Jer-Ming Chang

**Affiliations:** ^1^Department of General Medicine, Kaohsiung Medical University Hospital, Kaohsiung Medical University, Kaohsiung, Taiwan; ^2^Department of Internal Medicine, Kaohsiung Municipal Siaogang Hospital, Kaohsiung Medical University, Kaohsiung, Taiwan; ^3^Division of Nephrology, Department of Internal Medicine, Kaohsiung Medical University Hospital, Kaohsiung Medical University, Kaohsiung, Taiwan; ^4^Faculty of Medicine, College of Medicine, Kaohsiung Medical University, Kaohsiung, Taiwan; ^5^Research Center for Precision Environmental Medicine, Kaohsiung Medical University, Kaohsiung, Taiwan

**Keywords:** liver function parameters, incident diabetes mellitus, sex difference, follow-up, Taiwan Biobank

## Abstract

**Background:**

The prevalence of diabetes mellitus (DM) in Taiwan between 2017 and 2020 was 11.05%, which is higher than the global prevalence (10.5%). Previous studies have shown that patients with DM have higher liver enzyme levels than those without DM. However, it is unclear whether there are sex differences in the association between incident DM and liver function. Therefore, the aim of this longitudinal study was to investigate this issue in a large Taiwanese cohort.

**Methods:**

We identified 27,026 participants from the Taiwan Biobank, and excluded those with baseline DM (*n* = 2,637), and those without follow-up data on DM, serum fasting glucose or glycosylated hemoglobin A1c (*n* = 43). The remaining 24,346 participants (male: 8,334; female: 16,012; mean age 50.5 ± 10.4 years) were enrolled and followed for a median of 4 years.

**Results:**

Of the enrolled participants, 1,109 (4.6%) had incident DM and 23,237 (95.4%) did not. Multivariable analysis showed that high levels of glutamic-oxaloacetic transaminase (AST) (*p* < 0.001), glutamic-pyruvic transaminase (ALT) (*p* < 0.001), albumin (*p* = 0.003), α-fetoprotein (*p* = 0.019), and gamma-glutamyl transpeptidase (GGT) (*p* = 0.001) were significantly associated with incident DM in the male participants. In comparison, high levels of AST (*p* = 0.010), ALT (*p* < 0.001), albumin (*p* = 0.001) and GGT (*p* < 0.001), and low total bilirubin (*p* = 0.001) were significantly associated with incident DM in the female participants. There were significant interactions between total bilirubin and sex (*p* = 0.031), and GGT and sex (*p* = 0.011) on incident DM.

**Conclusion:**

In conclusion, liver function parameters were significantly associated with incident DM. Further, there were differences in the associations between the male and female participants.

## Introduction

Diabetes mellitus (DM) is a heterogeneous group of disorders characterized by hyperglycemia (1). Type 2 is the most common form of DM, and is cause by multiple pathophysiologic abnormalities. While insulin resistance in muscle/liver and β-cell failure remain the core defects, dysfunction of adipocytes, gastrointestinal tract, α-cells, kidney, and brain had also been found to be important in development of glucose intolerance in Type 2 DM population which form the concept of ominous octet (2). The International Diabetes Federation Diabetes Atlas estimated that the global prevalence of DM in people aged 20–79 years in 2021 was 10.5% (536.6 million people) (3). According to the Taiwan Health Promotion Administration, the prevalence of DM in Taiwan between 2017 and 2020 was 11.05%, which is higher than the global prevalence (10.5%) (4). Common risk factors for DM include overweight or obesity, high-risk race/ethnicity, history of cardiovascular disease, hypertension, physical inactivity, smoking and aging (5). The complications associated with DM include microvascular (diabetic nephropathy, neuropathy, and retinopathy), macrovascular (coronary artery disease, cerebrovascular disease), and miscellaneous types (6). The global diabetes-related health expenditure was estimated to be USD 966 billion in 2021 (3), highlighting the importance of detecting the potential risk factors for DM.

Liver function parameters could be classified to 3 main categories according to their functions: (1) Detection of hepatocellular injury such as glutamic-oxaloacetic transaminase (AST), glutamic-pyruvic transaminase (ALT) and gamma-glutamyl transpeptidase (GGT); (2) Liver's biosynthetic capacity such as albumin and α-fetoprotein (AFP); (3) Liver's capacity of transportation of the organic anions and to metabolize drugs such as total serum bilirubin (7). In the first category, detection of hepatocellular injury, marked elevations in ALT levels suggest hepatocellular injury such as viral hepatitis, ischemic liver injury and toxin-induced liver damage (8). AST is present in a wide variety of tissues including the heart, skeletal muscle, kidney, brain and liver, however it is not as sensitive as ALT to detect hepatocellular injury (9). In addition, an elevated GGT level may indicate liver diseases such as acute viral hepatitis, chronic hepatitis C and non-alcoholic fatty liver disease (NAFLD) (10), however it may also indicate the presence of non-liver diseases such as uncomplicated DM, acute pancreatitis and myocardial infarction (11). In the second category, liver's biosynthetic capacity, albumin is synthesized in the liver and it is one of the most important proteins in plasma. Since albumin is only synthesized in the liver, it is a useful indicator of hepatic function, and a decrease in albumin may indicate chronic liver disease or liver cirrhosis (12). An elevated level of AFP may also indicate liver injury and the early stages of chemical hepatocarcinogenesis (13), so it can be an indicator of hepatocellular carcinoma (HCC). In the last category, liver's capacity of transportation of the organic anions and to metabolize drugs, bilirubin is derived from the breakdown of hemoglobin, and an elevated level of the unconjugated form in the liver may suggest underlying liver disease or hemolysis (8). Since the liver performs a variety of functions, no single test is sufficient to completely evaluate its function (7).

Previous studies showed that patients with abnormal liver functions test were related to incident DM (14–17). However, it is unclear whether there are sex differences in the association between incident DM and liver function. Therefore, the aim of this longitudinal study was to investigate sex differences in the association between incident DM and liver function parameters (AST, ALT, albumin, AFP, total bilirubin, and GGT) in a large cohort derived from the Taiwan Biobank (TWB).

## Materials and methods

### TWB

The TWB is the largest biobank in Taiwan. It was established by The Ministry of Health and Welfare with the goals of promoting healthcare and preventing diseases, with a focus on the aging population in Taiwan. The TWB collects health-related data on ~200,000 healthy volunteers around Taiwan, as detailed below (18, 19). Ethical approval for the TWB was granted by the Ethics and Governance Council of the TWB and Institutional Review Board (IRB) on Biomedical Science Research, Academia Sinica, Taiwan.

The data collected by the TWB include body mass index (BMI), age, sex, and the presence of hypertension and DM. Fasting blood samples were obtained from all of the patients, and laboratory tests were conducted using an autoanalyzer (Roche Diagnostics GmbH, D-68298 Mannheim COBAS Integra 400). Overnight fasting blood and urine tests are also performed to collect data on uric acid, glucose, glycosylated hemoglobin A1c (HbA1c), triglycerides, total cholesterol, high-/low-density lipoprotein (HDL/LDL) cholesterol, estimated glomerular filtration rate (eGFR) [using the MDRD equation (20)], and liver function parameters (AST, ALT, albumin, AFP, total bilirubin, and GGT).

Data on blood pressure (BP) are also obtained, with the measurements made digitally by a TWB researcher three times with a 1–2-min gap between measurements. The participants are requested to avoid caffeine, exercise, and smoking for a minimum of 30 min prior to the measurements. Average systolic and diastolic BP measurements were analyzed in this study. Data on regular exercise, defined as ≥30 min of physical activity ≥3 times a week, were also recorded. This study was conducted according to the Declaration of Helsinki, and approved by the IRB of Kaohsiung Medical University Hospital (KMUHIRB-E(I)-20210058).

### Assessment of alcohol drinking and cigarette smoking history

All the participants also underwent a face-to-face interview with a researcher, during which they completed a questionnaire asking about alcohol drinking and cigarette smoking history. Subjects who had had smoked one cigarette or more per day for at least 1 year were defined as ever-smokers. Subjects who had drunk an alcoholic beverage, including beer, liquor, wine or Chinese herd wine, more than four times a week for at least 1 year were defined as ever drinkers.

### Participants

**Figure 1:**
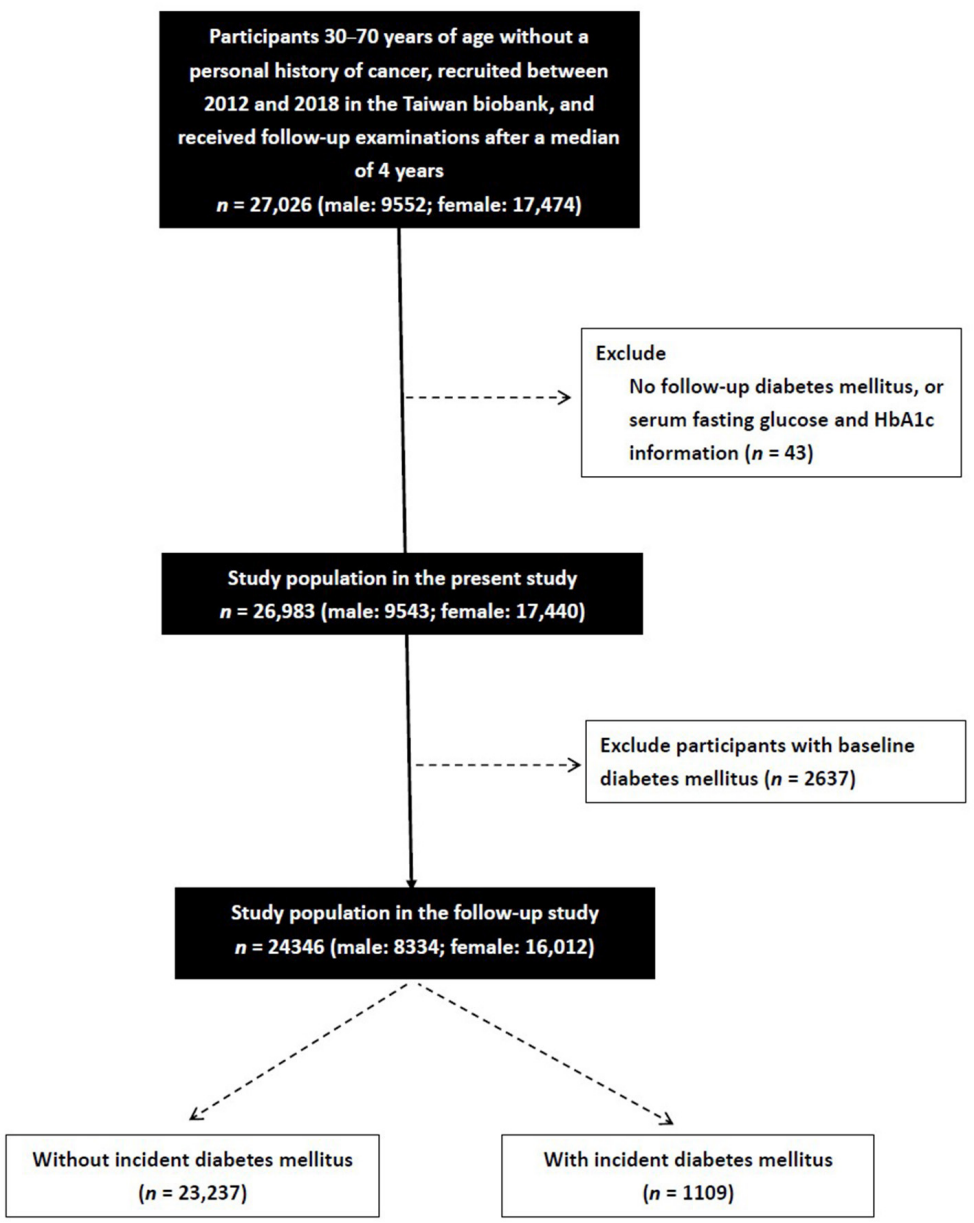
Flowchart of study population.

A total of 27,026 participants (male: 9,552; female: 17,474) were identified in the TWB. The participants who enroll in the TWB follow up after 2–4 years. Information, including a questionnaire, physical examination and blood examination, is collected upon first enrollment and second follow-up. Of whom those with no follow-up data on DM, serum fasting glucose or HbA1c (*n* = 43), and those with baseline DM (*n* = 2,637) were excluded. The remaining 24,346 participants were enrolled and followed for a median of 4 years (Figure 1). All of the enrolled participants gave written informed consent.

### Definition of incident DM

Participants with no past history of DM (self-reported) with a fasting glucose level < 126 mg/dL and HbA1c < 6.5% were defined as not having DM. Incident DM was defined as developing DM (self-reported, fasting glucose level ≥126 mg/dL or HbA1c ≥6.5%) during the follow-up period.

### Statistical analysis

Statistical analysis was done using SPSS version 19 (IBM Inc., Armonk, NY). Variables are shown as percentage or mean (±SD). Continuous variables were compared using the independent *t*-test, and categorical variables were compared using the chi-square test. Multivariable logistic regression analysis was used to examine associations between the development of incident DM and the studied liver function parameters (AST, ALT, albumin, AFP, total bilirubin, and GGT) in the male and female participants. An interaction *p* in logistic analysis was defined as: model disease (*y*) = *x*1 + *x*2 + *x*1 × *x*2 + covariates; where *x*1 × *x*2 is the interaction term, *y* = incident DM, *x*1 = sex, and *x*2 = the studied liver function parameters. The covariates were significant variables in univariable analysis. Receiver operating characteristic (ROC) curves were assessed the performance of the liver function parameters to identify incident DM, and areas under the ROC curves (AUCs) were used to assess their predictive ability. A two-tailed *p*-value < 0.05 was considered statistically significant.

## Results

Of the 24,346 enrolled participants (male: 8,334; female: 16,012; mean age, 50.5±10.4 years), 1,109 (4.6%) had incident DM and 23,237 (95.4%) did not. The incidence rates of DM were 5.7 and 4.0% in the males and females (*p* < 0.001), respectively.

### Characteristics of the with and without incident DM groups

The characteristics of the with and without incident DM groups are shown in Table 1. The incident DM group had a higher percentage of males, were older, had higher rates of hypertension, smoking, alcohol drinking, menstruation (in females), and higher systolic and diastolic BP, BMI, fasting glucose, HbA1c, triglycerides, total cholesterol, uric acid and LDL-cholesterol, and lower HDL-cholesterol and eGFR than the without incident DM group. With regards to the liver function parameters, the incident DM group had higher AST, higher ALT, higher albumin, lower total bilirubin and higher GGT. However, there was no significant difference in AFP.

**Table 1:** Comparison of clinical characteristics among participants without or with incident DM.

**Characteristics**	**Incident DM (–)** **(*n* = 23,237)**	**Incident DM (+)** **(*n* = 1,109)**	***p***
Age (year)	50.3 ± 10.4	54.7 ± 9.0	< 0.001
Male gender (%)	33.8	42.9	< 0.001
Hypertension (%)	10.2	25.5	< 0.001
Systolic BP (mmHg)	116.1 ± 17.2	125.3 ± 17.7	< 0.001
Diastolic BP (mmHg)	72.0 ± 10.8	76.3 ± 10.6	< 0.001
Smoking history (%)	24.5	31.1	< 0.001
Alcohol history (%)	2.7	4.7	< 0.001
Regular exercise habits (%)	47.6	48.7	0.493
BMI (kg/m^2^)	23.7 ± 3.4	26.1 ± 3.7	< 0.001
Menstruation in female (%)	47.9	27.8	< 0.001
**Laboratory parameters**			
Fasting glucose (mg/dL)	91.7 ± 7.3	101.5 ± 10.0	< 0.001
HbA1c (%)	5.6 ± 0.3	6.0 ± 0.3	< 0.001
Triglyceride (mg/dL)	107.2 ± 72.6	159.3 ± 134.5	< 0.001
Total cholesterol (mg/dL)	195.5 ± 34.8	203.1 ± 37.2	< 0.001
HDL-cholesterol (mg/dL)	55.2 ± 13.2	48.7 ± 11.3	< 0.001
LDL-cholesterol (mg/dL)	121.7 ± 31.1	128.9 ± 33.7	< 0.001
eGFR (mL/min/1.73 m^2^)	109.6 ± 25.0	106.0 ± 24.0	< 0.001
Uric acid (mg/dL)	5.4 ± 1.4	6.1 ± 1.5	< 0.001
**Liver function parameters**			
AST (U/L)	24.1 ± 10.9	28.3 ± 16.2	< 0.001
ALT (U/L)	22.4 ± 17.9	31.6 ± 26.5	< 0.001
Albumin (g/dL)	4.55 ± 0.23	4.58 ± 0.24	< 0.001
AFP (ng/mL)	3.32 ± 6.50	3.41 ± 5.75	0.636
Total bilirubin (mg/dL)	0.67 ± 0.28	0.64 ± 0.27	0.003
GGT (U/L)	22.6 ± 26.5	33.9 ± 40.8	< 0.001

DM, diabetes mellitus; BP, blood pressure; BMI, body mass index; HbA1c, glycosylated hemoglobin A1c; HDL, high-density lipoprotein; LDL, low-density lipoprotein; eGFR, estimated glomerular filtration rate; AST, glutamic-oxaloacetic transaminase; ALT, glutamic-pyruvic transaminase; AFP, α-fetoprotein; GGT, gamma-glutamyl transpeptidase.

### Comparisons of liver function parameters between the with and without incident DM groups in the male and female participants

The male participants with incident DM had higher AST, ALT, AFP, and GGT, but lower total bilirubin than the male participants without incident DM (Table 2). However, there was no significant difference in albumin. In addition, the female participants with incident DM had higher AST, ALT, albumin, and GGT, but lower total bilirubin than the female participants without incident DM. However, there was no significant difference in AFP.

**Table 2:** Comparison of clinical characteristics of the study participants classified by the presence of different sex and incident DM.

**Characteristics**	**Male (*****n*** = **8,334)**			**Female (*****n*** = **16,012)**		
	**Incidence of DM (–)** **(*****n*** = **7,858)**	**Incidence of DM (**+**)** **(*****n*** = **476)**	***p***	**Incidence of DM (–)** **(*****n*** = **15,379)**	**Incidence of DM (**+**)** **(*****n*** = **633)**	***p***
Age (year)	50.4 ± 11.0	53.9 ± 9.9	< 0.001	50.3 ± 10.1	55.3 ± 8.3	< 0.001
Hypertension (%)	14.3	29.0	< 0.001	8.2	22.9	< 0.001
Systolic BP (mmHg)	121.1 ± 16.0	126.4 ± 16.6	< 0.001	113.5 ± 17.2	124.4 ± 18.5	< 0.001
Diastolic BP (mmHg)	76.8 ± 10.4	79.2 ± 10.3	< 0.001	69.6 ± 10.2	74.1 ± 10.3	< 0.001
Smoking history (%)	57.9	64.3	0.006	7.5	6.2	0.201
Alcohol history (%)	6.8	10.5	0.002	0.7	0.3	0.446
Regular exercise habits (%)	48.4	45.6	0.228	47.2	51.0	0.061
Menstruation in female (%)	–	–	–	47.9	27.8	< 0.001
BMI (kg/m^2^)	24.8 ± 3.1	26.7 ± 3.6	< 0.001	23.2 ± 3.4	25.7 ± 3.7	< 0.001
**Laboratory parameters**						
Fasting glucose (mg/dL)	93.9 ± 7.2	102.8 ± 9.8	< 0.001	90.5 ± 7.0	100.5 ± 10.0	< 0.001
HbA1c (%)	5.57 ± 0.33	6.01 ± 0.29	< 0.001	5.54 ± 0.33	6.04 ± 0.28	< 0.001
Triglyceride (mg/dL)	127.3 ± 90.8	181.9 ± 168.5	< 0.001	96.9 ± 58.6	142.3 ± 95.4	< 0.001
Total cholesterol (mg/dL)	192.4 ± 33.8	197.6 ± 38.1	0.001	197.1 ± 35.2	207.3 ± 36.0	< 0.001
HDL-C (mg/dL)	48.8 ± 11.1	43.8 ± 8.9	< 0.001	58.5 ± 13.0	52.4 ± 11.6	< 0.001
LDL-C (mg/dL)	123.0 ± 30.8	126.3 ± 34.2	0.023	121.0 ± 31.3	130.9 ± 33.1	< 0.001
eGFR (mL/min/1.73 m^2^)	99.2 ± 19.8	96.9 ± 20.6	0.018	114.9 ± 25.7	112.9 ± 24.1	0.041
Uric acid (mg/dL)	6.5 ± 1.3	6.9 ± 1.5	< 0.001	4.9 ± 1.1	5.5 ± 1.1	< 0.001
**Liver function parameters**						
AST (U/L)	25.9 ± 11.3	30.1 ± 19.2	< 0.001	23.2 ± 10.6	26.9 ± 13.4	< 0.001
ALT (U/L)	27.4 ± 20.3	36.5 ± 31.5	< 0.001	19.9 ± 16.0	28.0 ± 21.3	< 0.001
Albumin (g/dL)	4.62 ± 0.23	4.63 ± 0.25	0.500	4.51 ± 0.22	4.54 ± 0.23	0.005
AFP (ng/mL)	3.14 ± 2.29	3.53 ± 8.60	0.007	3.41 ± 7.82	3.32 ± 1.52	0.255
Total bilirubin (mg/dL)	0.76 ± 0.32	0.73 ± 0.29	0.028	0.62 ± 0.24	0.58 ± 0.22	< 0.001
GGT (U/L)	29.9 ± 36.3	41.5 ± 51.5	< 0.001	18.9 ± 18.6	28.1 ± 29.1	< 0.001

Abbreviations are the same as in Table 1.

### Associations among liver function parameters with incident DM in the male and female participants

Multivariable logistic regression analysis was performed to examine associations among the liver function parameters with incident DM by sex (Table 3). In the male participants, after adjusting for age, hypertension, systolic and diastolic BPs, smoking and alcohol history, BMI, triglycerides, total cholesterol, LDL/HDL-cholesterol, eGFR and uric acid (significant variables in Table 1), high AST (per 1 U/L; odds ratio [OR], 1.013; 95% confidence interval [CI], 1.008–1.019; *p* < 0.001), high ALT (per 1 U/L; OR, 1.009; 95% CI, 1.006–1.012; *p* < 0.001), high albumin (per 1 g/dL; OR, 1.975; 95% CI, 1.254–3.110; *p* = 0.003), high AFP (per 1 g/mL; OR, 1.021; 95% CI, 1.003–1.039; *p* = 0.019), and high GGT (per 1 U/L; OR, 1.003; 95% CI, 1.001–1.005; *p* = 0.001) were significantly associated with incident DM. However, total bilirubin was not associated with incident DM in the male participants. In the female participants, after adjusting for the variables listed above for the male participants plus menstruation status, high AST (per 1 U/L; OR, 1.007; 95% CI, 1.002–1.012; *p* = 0.010), high ALT (per 1 U/L; OR, 1.007; 95% CI, 1.003–1.010; *p* < 0.001), high albumin (per 1 g/dL; OR, 2.018; 95% CI, 1.356–3.003; *p* = 0.001), low total bilirubin (per 1 mg/dL; OR, 0.515; 95% CI, 0.348–0.762; *p* = 0.001), and high GGT (per 1 U/L; OR, 1.006; 95% CI, 1.004–1.009; *p* < 0.001) were significantly associated with incident DM. However, AFP was not associated with incident DM in the female participants.

**Table 3:** Association of liver function parameters with incident DM using multivariable logistic regression analysis in different sex.

**Liver function parameters**	**Male (*****n*** = **8,334)**			**Female (*****n*** = **16,012)**			**Interaction *p***
	**Multivariable** ^*^			**Multivariable** ^#^			
	**OR**	**95% CI**	***p***	**OR**	**95% CI**	***p***	
AST (per 1 U/L)	1.013	1.008–1.019	< 0.001	1.007	1.002–1.012	0.010	0.126
ALT (per 1 U/L)	1.009	1.006–1.012	< 0.001	1.007	1.003–1.010	< 0.001	0.315
Albumin (per 1 g/dL)	1.975	1.254–3.110	0.003	2.018	1.356–3.003	0.001	0.586
AFP (per 1 g/mL)	1.021	1.003–1.039	0.019	0.992	0.967–1.016	0.503	0.069
Total bilirubin (per 1 mg/dL)	0.941	0.688–1.288	0.706	0.515	0.348–0.762	0.001	0.031
GGT (per 1 U/L)	1.003	1.001–1.005	0.001	1.006	1.004–1.009	< 0.001	0.011

Values expressed as odds ratio (OR) and 95% confidence interval (CI). Abbreviations are the same as in Table 1.

^*^Adjusted for age, hypertension, systolic and diastolic blood pressures, smoking and alcohol history, body mass index, triglyceride, total cholesterol, HDL-cholesterol, LDL-cholesterol, eGFR and uric acid (significant variables in Table 1).

^#^Adjusted for age, hypertension, systolic and diastolic blood pressures, smoking and alcohol history, body mass index, triglyceride, total cholesterol, HDL-cholesterol, LDL-cholesterol, eGFR, uric acid and menstruation status (significant variables in Table 1).

### Interactions among liver function parameters and sex on incident DM

Significant interactions were found between total bilirubin and sex (*p* = 0.031), and GGT and sex (*p* = 0.011) on incident DM (Table 3).

### Performance and predictive ability of the liver function parameters to identify incident DM

The AUCs of the liver function parameters to incident DM in the male and female participants are shown in Table 4. In the male participants, GGT had the highest AUC (0.631), followed by ALT (0.617), AST (0.570) and total bilirubin (0.472). Albumin and AFP were not significantly associated with incident DM. In the female participants, GGT also had the highest AUC (0.701), followed by ALT (0.679), AST (0.614), total bilirubin (0.441), AFP (0.558) and albumin (0.540).

**Table 4:** Area under curve of liver function parameters for incident DM of different sex.

**Liver function parameters**	**Male (*****n*** = **8,334)**			**Female (*****n*** = **16,012)**		
	**AUC**	**95% CI**	***p***	**AUC**	**95% CI**	***p***
AST	0.570	0.542–0.598	< 0.001	0.614	0.591–0.636	< 0.001
ALT	0.617	0.591–0.644	< 0.001	0.679	0.658–0.699	< 0.001
Albumin	0.515	0.488–0.542	0.273	0.540	0.517–0.563	0.001
AFP	0.516	0.490–0.542	0.230	0.558	0.536–0.579	< 0.001
Total bilirubin	0.472	0.445–0.499	0.039	0.441	0.419–0.464	< 0.001
GGT	0.631	0.605–0.656	< 0.001	0.701	0.682–0.719	< 0.001

Values expressed as area under curve (AUC) and 95% confidence interval (CI). Abbreviations are the same as in Table 1.

## Discussion

In this study, we investigated sex differences in the associations between incident DM and liver function parameters after a median 4-year follow-up period. We found that high AST, ALT, albumin and GGT were associated with incident DM in both sexes. However, high total bilirubin was only associated with incident DM in the females, and high AFP was only associated with incident DM in the males. Further, we found significant interactions between total bilirubin and GGT and sex on incident DM.

The first important finding of this study is that high AST, ALT, albumin and GGT were associated with incident DM in both sexes. ALT is an enzyme primarily found in the liver, and it is more closely related to hepatocellular injury or fat deposition (21). Although AST is present in the liver, it is also present in other organs including cardiac and skeletal muscles, kidneys and brain, and it is less specific for hepatic damage then ALT (22). The AST to ALT ratio has been use to discern the different etiologies of hepatic injury (23). Previous studies have reported associations between ALT and type 2 DM (24, 25). Ohlson et al. (24) reported that an increase in ALT was associated with a higher relative risk of incident DM in middle-aged Swedish men. Vozarova et al. (25) analyzed 451 Pima Indians, and found that high ALT was an independent predictor of incident type 2 DM after adjusting for age, sex, body fat, insulin sensitivity and acute insulin response. In addition, Goessling et al. (26) reported that both ALT and AST were associated with a greater risk of incident DM after adjusting for baseline blood glucose and changes in weight. Moreover, they also found that only ALT was associated with incident DM when using normal values in the analysis (26). Previous studies have also shown a close relationship between NAFLD with type 2 DM (27). In patients with NAFLD, increases in ALT and AST by more than 2–5 times the normal limit and an AST/ALT ratio < 1 have consistently been reported, possibly due to the effect of hepatocyte damage (23, 28). Another possible link between DM and NAFLD may be due to insulin resistance and visceral fat deposition, both of which can affect the regulation of lipoprotein and glucose. Under conditions of increasing insulin resistance, the downregulation of lipolysis by insulin can lead to further adipose deposition on hepatocytes, thereby further inducing steatosis (21). Taken together, these explanations may partially explain our findings of associations between high AST and ALT with incident DM.

Another key finding is that we found an association between high albumin and incident DM in both sexes. Serum albumin is mostly produced by the liver, and it accounts for around half of all human plasma proteins. Albumin regulates the oncotic pressure of blood and transports many small molecules (29). A decrease in liver function may result in hypoalbuminemia, which can lead to general edema, fluid loss to the third space, and hyperlipidemia (30). Kunutsor et al. (16) found a nearly linear independent positive association between type 2 DM and serum albumin. In addition, Bae et al. (31) reported that increased serum albumin was positively associated with insulin resistance, but that it was not an independent factor for incident DM. In contrast, Schmidt et al. (32) found that a low serum albumin level was associated with an increased risk of type 2 DM among 12,330 men and women aged from 45 to 64 years. Chang et al. (33) also found that a decrease in albumin level was associated with an increased risk of type 2 DM, and the authors attributed this result to a decrease in hepatic albumin synthesis and increase in glycated albumin, which may increase oxidative stress and inflammation. Although we found that high albumin was associated with incident DM, further studies are needed to clarify the underlying mechanisms.

High GGT was associated with incident DM in both sexes in this study. GGT is an enzyme which catabolizes extracellular glutathione, and it has been widely used as a parameter of liver function. GGT is metabolized in the epithelial cells of the intrahepatic duct, which play an important role in glutathione equilibrium (34). An elevation in GGT has been linked to greater oxidative stress due to increasing glutathione catabolism (antioxidant agent), which may lead to ß-cell dysfunction and a decrease in insulin activity (35). In a cross-sectional study of 7,976 participants from the National Health and Nutrition Examination Survey from 1999 to 2002, Sabanayagam et al. (36) found that higher serum GGT levels were positively associated with DM, independent of alcohol consumption, BMI, hypertension and other confounders. Fraser et al. (14) conducted a meta-analysis of 18 prospective population-based studies, and also found a positive association between GGT and incident DM. Kunutsor et al. (15) conducted another meta-analysis of 24 cohort studies with 177,307 participants focusing on the nature of the dose-response relationship between GGT and incident DM, and found a non-linear association between GGT and the risk of type 2 DM in both sexes. Moreover, the interactions between GGT and sex on incident DM were also statistically significant. We found that high GGT was more strongly associated with incident DM in the females than in the males in our study. This could be explained from several aspects. Previous studies have shown that GGT level may be affected by estrogen, menopausal stage, and even the use of oral contraceptives. Nilssen and Førde (37) found that starting to use oral contraceptives and menopause were associated with an increase in GGT level, and Serviddio et al. (38) found that estrogen was negatively associated with glutathione. As a catalyzer of glutathione, GGT may also be positively related to the level of estrogen. Moreover, Wang et al. (39) found that the association between elevated GGT and cardiovascular mortality was stronger in females than in males. Hozawa et al. (40) also found a strong positive association between elevated GGT and cardiovascular mortality among Japanese women, but not in men. Both studies concluded that their findings were due to the high percentage of alcohol consumption in men, which may also affect circulating oxidative stress. Female hormones and excessive alcohol consumption in men may play an important role, however the mechanisms underlying sex differences in the association between GGT and incident DM are still not fully understand, and further research is needed.

Another important finding of this study is the association between high AFP with incident DM in males but not in females. AFP belongs to the family of serum albumins produced by the yolk sac and fetal liver during fetal development (41). It is usually at the highest level in infants, and then decreases to normal range before 1 year of age (42). It is used to screen for specific malignancies such as HCC (43) and developmental abnormalities from maternal blood or amniotic fluid (44) in current clinical practice. Moreover, the incidence and mortality rates of HCC are higher in people with DM (45). Obesity is an important risk factor shared between DM and HCC. The pathogenesis is associated with lipid peroxidation, which can lead to an increase in free radical oxidative stress (46) and mutations of p53 tumor suppressor (47), which can both lead to hepatic carcinogenesis (48). Moreover, obesity may cause insulin resistance with hyperinsulinemia, further leading to an increase in insulin-like growth factor-1 which then promotes proliferation and inhibits apoptosis through receptor-mediated pathways, resulting in hepatic carcinogenesis (49). Since the prevalence of HCC is about 2–3 times higher in males compared with females (43), this may explain why a higher AFP level was only associated with incident DM in the males and not females. Another possible explanation for the relationship between AFP and incident DM may be related to metabolic syndrome, which is related to the development of DM, cardiovascular disease, and NAFLD. A possible mechanism for the association between metabolic syndrome and elevated AFP may be due to insulin resistance and fatty liver disease. Both are usually accompanied with each other and they may influence the hemostasis of hepatic glucose, further leading to a chronic inflammatory status of the liver (50).

The last important finding of this study is that low total bilirubin was associated with incident DM in the females but not in the males. Bilirubin is traditionally considered to be derived from the breakdown of hemoglobin *via* normal catabolic pathways, and it is clinically related to jaundice (51). However, recent studies have shown that it is also a potential antioxidant which is inversely related to a lower prevalence of oxidative stress-mediated diseases (52). In a meta-analysis of cross-sectional studies, Nano et al. (53) found an inverse association between bilirubin level and type 2 DM. Several studies have also revealed similar results of an inverse relationship between serum bilirubin level and incident type 2 DM (17). In our study, we found that bilirubin level was only significantly inversely related to incident DM in the females but not in the males. A possible explanation for this finding may be related to different interactions between hormones and bilirubin metabolism in males and females. Kao et al. (54) found that estrogen may facilitate bilirubin metabolism in a regenerating liver by enhancing the expression of cytochrome (CYP2A6). Moreover, Muraca et al. found that hepatic bilirubin UDP-glucuronosyltransferase activity, an enzyme that catalyzes the conjugation of bilirubin and plays an important role in bilirubin excretion, was higher in female than in male rats, but that decreased enzyme activity in female rats and increased activity in male rats were noted after gonadectomy. Therefore, the excretion of bilirubin decreased in the female rats but increased in the males rats after gonadectomy (55), which may partially explain our findings. Another possible mechanism of differences in the association between bilirubin and incident DM between sex maybe related to difference of heme oxygenase (HO) expression between male and female. HO is an enzyme play an important role of heme catabolism to produce biliverdin, and carbon monoxide and eventually increase bilirubin which is the end product of heme catabolism (56). The HO system is related to antioxidant and anti-apoptotic because of its byproducts, bilirubin/biliverdin and carbon monoxide (57). HO-1 is induced by oxidant stress and plays a crucial role of antioxidant in diabetes by improving insulin sensitivity, reduces adipose tissue volume, and causes adipose tissue remodeling (58). An animal study of rats found that trauma and hemorrhage induced a twofold increase in hepatic HO-1 expression in proestrus females compared with males (59). This may explain the mechanism of differences in the association between bilirubin and incident DM between sex.

The strengths of this study include that the analysis involved a large cohort, and the comprehensive follow-up data to analyze sex differences in the association between liver function and incident DM. Despite these strengths, several limitations should be noted. First, information on the presence/absence of fatty liver, dietary issues, and certain medications (ex. renin-angiotensin-aldosterone system blockers, and statins) which could affect the development or prevention of incident DM is not available in the TWB, which may have resulted in underestimation of the association between liver function and incident DM. In addition, information on factors which could lead to incident DM such as proteinuria is also not available in the TWB. Another limitation is that we only enrolled participants of Han ethnicity residing in Taiwan, and thus our findings may not be generalizable to other ethnicities/areas. Finally, sample bias may have been introduced, as only around 25% of participants in the TWB return for follow-up evaluations.

In conclusion, liver function parameters were significantly associated with incident DM. Further, there were differences in the associations between the male and female participants.

## Data availability statement

The raw data supporting the conclusions of this article will be made available by the authors, without undue reservation.

## Ethics statement

This study was conducted according to the Declaration of Helsinki, and approved by the IRB of Kaohsiung Medical University Hospital (KMUHIRB-E(I)-20210058). The patients/participants provided their written informed consent to participate in this study.

## Author contributions

Conceptualization, methodology, validation, formal analysis, writing—review and editing, supervision, and data curation: Y-KC, P-YW, J-CH, S-CC, and J-MC. Software, investigation, resources, project administration, and funding acquisition: S-CC. Writing—original draft preparation: Y-KC and S-CC. Visualization: S-CC and J-MC. All authors have read and agreed to the published version of the manuscript.
